# Assessment of Whole-Genome Regression for Type II Diabetes

**DOI:** 10.1371/journal.pone.0123818

**Published:** 2015-04-17

**Authors:** Ana I. Vazquez, Yann C. Klimentidis, Emily J. Dhurandhar, Yogasudha C. Veturi, Paulino Paérez-Rodríguez

**Affiliations:** 1 Department of Epidemiology and Biostatistics, Michigan State University, East Lansing, MI, United States of America; 2 Mel and Enid Zuckerman College of Public Health, Division of Epidemiology and Biostatistics, University of Arizona, Tucson, AZ, United States of America; 3 Department of Health Behavior, School of Public Health, University of Alabama at Birmingham, Birmingham, AL, United States of America; 4 Department of Biostatistics, School of Public Health, University of Alabama at Birmingham, Birmingham, AL, United States of America; 5 Colegio de Postgraduados, Montecillo, Edo. de Mexico, Mexico; National Taiwan University, TAIWAN

## Abstract

Lifestyle and genetic factors play a large role in the development of Type 2 Diabetes (T2D). Despite the important role of genetic factors, genetic information is not incorporated into the clinical assessment of T2D risk. We assessed and compared Whole Genome Regression methods to predict the T2D status of 5,245 subjects from the Framingham Heart Study. For evaluating each method we constructed the following set of regression models: A clinical baseline model (**CBM**) which included non-genetic covariates only. CBM was extended by adding the first two marker-derived principal components and 65 **SNPs** identified by a recent GWAS consortium for T2D (**M-65SNPs**). Subsequently, it was further extended by adding 249,798 genome-wide SNPs from a high-density array. The Bayesian models used to incorporate genome-wide marker information as predictors were: Bayes A, Bayes Cπ, Bayesian LASSO (**BL**), and the Genomic Best Linear Unbiased Prediction (**G-BLUP**). Results included estimates of the genetic variance and heritability, genetic scores for T2D, and predictive ability evaluated in a 10-fold cross-validation. The predictive AUC estimates for CBM and M-65SNPs were: 0.668 and 0.684, respectively. We found evidence of contribution of genetic effects in T2D, as reflected in the genomic heritability estimates (0.492±0.066). The highest predictive AUC among the genome-wide marker Bayesian models was 0.681 for the Bayesian LASSO. Overall, the improvement in predictive ability was moderate and did not differ greatly among models that included genetic information. Approximately 58% of the total number of genetic variants was found to contribute to the overall genetic variation, indicating a complex genetic architecture for T2D. Our results suggest that the Bayes Cπ and the G-BLUP models with a large set of genome-wide markers could be used for predicting risk to T2D, as an alternative to using high-density arrays when selected markers from large consortiums for a given complex trait or disease are unavailable.

## Introduction

While the Human Genome Project provides a detailed description of genetic variation, the causal genes for many diseases are yet to be found. Although some variants have been found to be directly causal and to increase the risk of disease, the significant variants associated with complex traits have been found to explain only a small percentage of the phenotypic variation. This problem has been referred to as the ‘missing heritability’ of complex traits and diseases [1,2]. However, recent studies show that by using information from hundreds of thousands of loci with Whole Genome Regression (**WGR**), all the heritability in family-based data[3,4] and half of the expected heritability in complex traits can be explained [4–6].

Of late, there is an increasing interest in estimating the variance in complex traits that is explained by molecular markers, with the ultimate goal of obtaining accurate estimates of individual genetic predisposition to disease. With Whole Genome Prediction (**WGP**), genetic risk is modeled using thousands of (small-effect) loci concurrently [5,7]. The foundational idea of these whole-genome methods was published by Meuwissen et al. and it has since brought about a revolution in the animal and plant breeding communities in both academia and industry [8–10]. Modeling genetic risk for human disease by regressing high-density-SNP arrays on phenotypes is feasible with the use of penalized and Bayesian variable selection [11,12] and shrinkage estimation methods [13–15]. Since 2001, WGR has been extensively used [5,6,16–18] to estimate genetic parameters [3,6,19] and, more recently, to predict genetic risk [4,18,20].

In samples of related humans, an increment in the number of markers used by the model increases the genetic variance explained and monotonically increases the prediction accuracy [4]. However, in samples of unrelated humans, while variance explained can reach up to 50% of the genetic heritability [5,7], there is an optimum number of SNPs at which prediction accuracy is maximized [21]. Therefore, in unrelated samples of humans, relatively poor prediction accuracy of disease risk is achieved. The span of linkage disequilibrium (LD) is much shorter in humans compared to domestic agricultural species (e.g., cattle [22]), thus genetic markers cannot correctly estimate genomic relationships and the statistical model cannot separate genetic signal from random variation [21]. Consequently, empirical studies show that while the prediction R-squared in validation samples (for human height) is approximately 0.3 in a family-based sample, it is only about 0.03–0.05 in unrelated individuals [21]. In summary, in family-based samples, the predictive ability is higher if the model is informed by relatives who share large sections of the chromosome with the individuals to be predicted [4,18]. There, a SNP that is distant from a causal locus can still be highly informative of the genetic risk of disease. In short, prediction in related and unrelated individuals are two different problems. Thereby, for complex human traits in unrelated subjects, it may be important to reduce the noise from the genotype by targeting regions of causal loci. Whole Genome Regressions are a large family of methods that can either differentiate genetic regions or weight the entire genome equally. However, how different WGRs work for prediction of unrelated subjects has not been completely addressed yet.

WGRs differ in the priors assigned to the marker effects and in their ability to perform selection and shrinkage of predictors. Some WGRs (e.g., G-BLUP) have an underlying assumption that all predictors have some small effect, with genetic risk being determined by a very large number of variants. This implies that the trait (e.g. human height) has a highly complex genetic architecture [5]. Other priors from the thick tailed family (e.g., the scaled-t, or the double-exponential) have, relative to the Gaussian prior, higher mass at zero and thicker tails; examples include Bayes A [16] and the Bayesian LASSO (BL) [15]. Finally, finite-mixture priors assign a certain prior probability for the effects to be equal to zero. These priors—for example, Bayes C*π* [12,23]—induce variable selection and shrinkage simultaneously and work best for traits whose genetic architectures include regions that do not contribute to genetic risk at all.

In this article we aim to evaluate several Bayesian models, including BL, Bayes A, Bayes C*π*, and G-BLUP that perform differential shrinkage and variable selection. We focus on Type 2 Diabetes Mellitus (**T2D**) since it is the fastest growing chronic disease in the developing world [24]. A complex interaction between lifestyle factors and genetics (h^2^ between 0.25–0.70 in family and monozygotic twins) plays a large role in the development of T2D [25–29]. Additionally, for T2D, several studies report highlights of the genetic architecture of T2D by uncovering several SNP variants [30–32], and recently 65 SNPs have been associated with T2D [30]. In our study, we included a benchmark model with these well-established SNPs to evaluate the performance of the Bayesian methods.

## Materials and Methods

### Ethics Statement

The FHS obtained informed consent from the participants to use their clinical records for research purposes such as this study. Additionally, we obtained the data from dbGap, where data is de-identified before being distributed to other researchers.

### Data

#### Phenotypes

The data set consists of 5,245 participants (2,381 males and 2,864 females) from the Framingham Heart Study, which has collected longitudinal phenotypic information in several generations of families [33,34]. Subjects in this study have been characterized every other year from adulthood to death on risk factors, outcomes of physical exams, and disease status. T2DM was defined as having blood sugar ≥ 126 mg/dL, at any exam, or having ever taken anti-diabetic medication. The studies used have the dbGaP (database of Genotypes and Phenotypes) accession number pht000040.v4.p7, pht000041.v4.p7 and pht000311.v5.p7. Participants included in our study belong to the Original cohort (*n* = 1,498), and the cohort that is comprised of their Offspring (*n* = 3,747). We excluded subjects from the third generation cohort because their follow up time is still too short. Data and material distributions from this study are made in accordance with the individual consent history of each participant and the current study has been approved by the Internal Review Board of University of Alabama at Birmingham (IRB Protocol Number: X090720002).

#### Genomic Information

All subjects were genotyped for single nucleotide polymorphisms (SNPs) with the Affymetrix 500K chip. Details on the genotyping method are described at the Framingham SHARe at the NCBI dbGaP website (http://www.ncbi.nlm.nih.gov/projects/gap/cgi-bin/study.cgi?study_id=phs000007.v3p2). We removed markers with minor allele frequency< 0.05 or with more than 10% missing genotypes. After edition, we randomly reduced the platform to approximately half of the SNPs (*p* = 249,798) to attenuate computational demand. This platform included 20 SNPs of the 65 SNPs previously published. Pre-analysis including and excluding the 65 markers suggests that including or excluding them in WGR does not vary WGR results.

### Statistical Methods

The outcome ***y*** = {*y*_*i*_} was defined as presence (*y*_*i*_ = 1) or absence (*y*_*i*_ = 0) of T2D (blood sugar > 126 mg/dL, or having ever taken an anti-diabetic medication) during the follow up time of the FHS. We assessed several models including WGRs with various types of Bayesian approaches that differ in the selection and shrinkage applied to the marker effects. This section is organized as follows: (a) the description of the probit link connecting the response variable (diabetes presence or absence) with a linear predictor (*η*_*i*_); (b) the sequence of models developed; (c) the Bayesian statistical models evaluated, (d) estimates of genetic effects associated to markers, and (e) model evaluation tools.

#### a) Probit Link

Let *y*_*i*_ be the random variable that denotes the presence or absence of diabetes and define *ϑ*_*i*_ (i.e. the probability of having diabetes) as *ϑ*_*i*_ = *P*(*y*_*i*_ = 1|***x***_*i*_). It follows that *y*_*i*_ is distributed as a Bernoulli random variable with probability of success given by *ϑ*_*i*_ (*ϑ*_*i*_) a subject-specific Bernoulli parameter). In probit regression, the probability of success depends on a set of covariates (***x***_*i*_′*s*) and is modeled as,

$$\vartheta_{i}=\Phi \left(\eta_{i}\right),$$

where Φ(⋅) is the cumulative distribution function of the standard normal distribution, and (*η*_*i*_) is a model dependent linear predictor that will be described next.

#### b) Sequence of statistical models

*Covariates baseline model* (**CBM**). The CBM included non-genetic covariates only. The linear predictor for CBM is

$$\eta_{i}=\alpha_{0}+\alpha_{1}s_{i}+\alpha_{2}c_{i}+\alpha_{3}l_{i}$$

Where *η*_*i*_ is represented as the sum of an intercept(*α*_0_), plus a regression on the ‘fixed effects’ of sex (*s*_*i*_, as dummy variable), cohort (*c*_*i*_, a dummy variable indicating whether participants were from the Original or Offspring cohort), the age at last contact or death (*l*_*i*_, ranging from 34 to 104) to control for different exposure times or observational periods, and ***α*** = (*α*_1_,…, *α*_3_)′, are the corresponding regression coefficients. The sample from FHS includes subjects from two cohorts and each cohort starts at a different year, a few of the measures could have different protocols and a different data collection team. We included cohort information in the models to correct for these factors.

*65-SNP Model* (**M-65SNP**). The CBM model was first extended by adding 2 marker-derived principal components (*PC1* and *PC2*) and 65 SNPs that have been consistently associated with T2DM. The PCs were derived from 1,000 European-ancestry informative markers reported by [35]. This model is a second benchmark or baseline model to compare with WGR. The linear predictor could then be expressed as,

$$\eta_{i}=\alpha_{0}+\alpha_{1}s_{i}+\alpha_{2}c_{i}+\alpha_{3}l_{i}+\alpha_{4}PC1_{i}+\alpha_{5}PC2_{i}+\sum_{j=1}^{65}\gamma_{j}x_{ij},$$

where *α*_4_ and *α*_5_ are regression coefficients associated with PCs 1 and 2 respectively; *x*_*ij*_ is the genotype of the *i* ^th^ individual (*i* = 1,…, 5,245) at the *j* ^th^ marker (*j* = 1,…,65), expressed as the count of one of the two alleles *x*_*ij*_ ∈ {0,1,2}, for the imputed SNPs *x*_*ij*_ ∈ [0,2] (a real number) and the ***γ*** = {*γ*_*i*_}’s are marker effects. When absent in the platform, these SNPs were imputed with IMPUTE2 with 1,092 subjects from the 1,000 Genomes data as reported previously [36–38].

#### c) Bayesian models for Whole Genome Regressions

Subsequently, we evaluated several WGRs using the CBM as the base and included the genomic effects (*u*_*i*_) modeled with whole-genome markers. These were comprised of a high density array of *p* = 249,798 SNPs and were regressed on a function of the phenotype evaluated in this study. SNP effects were included in the models using either Bayes A, Bayes C*π*, Bayesian LASSO (BL), and G-BLUP. The linear predictor for these models could be written in general as,

$$\eta_{i}=\alpha_{0}+\alpha_{1}s_{i}+\alpha_{2}c_{i}+\alpha_{3}l_{i}+\alpha_{4}PC1_{i}+\alpha_{5}PC2_{i}+u_{i}$$

In addition to the joint conditional probability of the data, given the unknown coefficients, the prior density of the unknowns was flat for ***α***, i.e. *p*(***α***) ∝ 1. This yields estimates of effects comparable to those obtained with maximum likelihood. The genomic effect term *u*_*i*_ is different in every one of the Bayesian models evaluated. The definition of *u*_*i*_ and its prior probability completes the Bayesian model. We describe them below for each Bayesian model evaluated.

*Bayes A (* ***BA*** *)*. In Bayes A models [16], $u_{i}=\sum_{j=1}^{p}x_{ij}\beta_{ij}$ and the prior density of the SNPs effects is assumed to follow a t distribution, *T*(*β*_*j*_|*df*_*β*_, *S*_*β*_), (*j* = 1,…,65), which could be re-expressed as $\int N\left(\beta_{j}|0,\sigma_{\beta_{j}}^{2}\right)\chi^{-2}\left(\sigma_{\beta_{j}}^{2}|df_{\beta_{j}},S_{\beta_{j}}\right)\partial \sigma_{\beta_{j}}^{2}$ where $\sigma_{\beta_{j}}^{2}$ is the variance of the marker effects corresponding to the *j* ^*th*^ position; see [39]. Thus, the conditional distribution of marker effects *β*_*j*_ is normal with mean 0 and variance $\sigma_{\beta_{j}}^{2}$, at the next level of hierarchy we assigned a scaled-inverse chi squared distribution to the variance of marker effects. The corresponding hyper-parameters for the scaled-inverse chi-squared distribution were set according to the rules given in [40] and implemented in the BGLR package.

*Bayes C* π *(* ***BC*** *)*. In Bayes C models [12], $u_{i}=\sum_{j=1}^{p}x_{ij}\beta_{ij}$ and here the prior for the marker effects is a two component mixture. One of the components is a point of mass at zero and the other component is a normal distribution. The prior for the marker effect for this model can be written as,

$$\text{p}\left(\beta_{\text{j}}|\pi ,\sigma_{\beta }^{2}\right)=\pi \times 1\left(\beta_{\text{j}}=0\right)+\left(1-\pi \right)\times \text{N}\left(\beta_{\text{j}}|0,\sigma_{\beta }^{2}\right)$$

where π is the proportion of markers with non-null effects and the prior assigned to π was *Beta*(*p*_0_, *π*_0_) (see[41]). We assigned a scaled-inverse chi-squared distribution to $\sigma_{\beta }^{2}$, the corresponding hyper parameters were set using the rules given in de los Campos et al. (2013).

*Bayesian LASSO (* ***B-LASSO*** *)*. In the Bayesian LASSO [15], $u_{i}=\sum_{j=1}^{p}x_{ij}\beta_{ij}$ and the prior density of the SNPs effects can be expressed as $N\left(\beta_{j}|0,\tau_{j}^{2}\right)$, where the prior distribution of $\tau_{j}^{2}$ is exponential, i.e. $Exp\left(\tau_{j}^{2}\lambda^{2}\right)$ and the prior density assigned to *λ* ^2^ is a gamma distribution, *G*(*λ* ^2^ | *δ*_1_, *δ*_2_), with the hyper-parameter rate set to 0.0001 and shape 0.55; (For further details on priors for this model see [42]).

*G-BLUP*. In this model [43], ***u*** = {*u*_*i*_} is a random effect in the regression which distribution is $N\left(u|0,G\sigma_{u}^{2}\right)$ where ***G*** = {*G*_*ii*′_} is an *n* × *n* matrix of relationships based on the *p* SNP genotypes such that,

$$G_{ii^{\prime }}=\frac{1}{n}\times \frac{\sum_{j=1}^{p}\left(x_{ij}-2q_{j}\right)\left(x_{i^{\prime }j}-2q_{j}\right)}{2\sum_{j=1}^{p}q_{j}\left(1-q_{j}\right)},$$

where *q*_*j*_ is the estimated *j* ^*th*^ allele frequency; and $\sigma_{u}^{2}$ is an ‘additive’ genetic variance parameter. We assigned a scaled-inverse chi-squared distribution to $\sigma_{\beta }^{2}$, the corresponding hyper parameters were set using the rules given in de los Campos et al. (2013). The marker effects were obtained with the equivalent Bayesian Ridge Regression model, as described elsewhere [40].

The parameters of the above-described model were estimated in a Bayesian framework using the BGLR package[41,44] in R [45]. Priors used were relatively non influential [41]. We used 40,000 MCMC iterations with 15,000 samples taken as burn in. Convergence was assessed by visual inspection of the trace plots, e.g. S1 Fig and S2 Fig.

### Genomic Heritability

In the models described above, narrow sense heritability in the liability scale is defined as the ratio of the genetic variance to the total variance. The residual variance is fixed at one, thus the narrow sense heritability is $h_{G}^{2}=\frac{\sigma_{u}^{2}}{\sigma_{u}^{2}+1}$, since the residual variance is set to one as defined elsewhere for binary traits[46,47]. The genomic heritability is interpreted as the proportion of inter-individual differences at risk for T2D that can be explained by regression on common SNPs.

### Model Evaluation

The covariates included in all models were selected based on significance. This evaluation was done with the generalized linear model (*glm* function) from the R base package [45]. Models were compared based on effect estimates from published GWAS. We present distribution of effects and scatter plots of these estimates for each model. Additionally, we assessed the models' prediction accuracy using a 10-fold cross-validation. Since Framingham is a family based study, we randomized and assigned entire families, according to the pedigree, to folds such that when the model is trained, neither the subject to be predicted nor the subjects in the same fold—which include all subjects in one family—are used to fit the predictive model. The testing sets of the cross-validation yielded predictions of risk scores $\left\{\hat{\eta_{i}}\right\}$, which were derived without using the *i* ^*th*^ observation or any relative of the *i* ^*th*^ observation. AUC was computed using the pairs of points that included the presence/absence of diabetes and the risk score was predicted using cross validation $\left\{y_{i},\hat{\eta_{i}}\right\}$. Since realization of diabetes (*y*_*i*_) is a binary response (0/1), it is more appropriate to report results in terms of false positive rate and Area Under the Receiver Operating Characteristic Curve (**AUC**, see [48]). We estimated the former statistics using the R package ROCR [49].

## Results

### Descriptive Statistics

**Fig 1:**
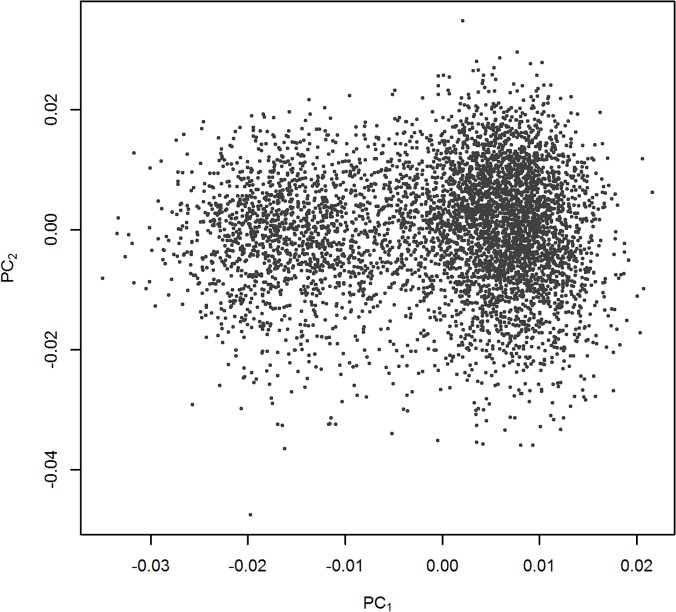
Principal Components 1 and 2, derived from 1,000 ethnicity informative SNPs for European origin.

Participants in the two cohorts that were included in this study were born between 1890 and 1968, and the age at either last contact or death was 74±12 (mean ± standard deviation). The total number of T2D cases was 939 out of the 5,245 subjects; since FHS is an ongoing study, many study participants do not have diabetes yet. The incidence of diabetes in participants with last contact at age <65 was 8%, while it was 26% for participants with last contact at age > 83 years. The 939 cases showed a first record of diabetes at 63±24 years of age [50]. The proportion of subjects in the population with diabetes between cohorts was 30.2% in the Original cohort and 13.0% in the Offspring cohort (paternal and offspring generations, respectively). This difference reflects the shorter observational time of the Offspring cohort, whose subjects have an age at the last contact time (or death time) of 69±10, whereas this age is 87±8 for the Original cohort. The proportion of people that had diabetes was 20.5% in males (45% of the sample) and 15.7% in females (55% of the sample). This difference in proportions is in accordance with what has been observed in the literature where males have a higher incidence of diabetes [50]. In the study we also included principal components derived from ethnicity informative SNPs to account for population structure; Fig 1 shows a scatter plot with the ethnicity-informed marker-derived PC 1 and 2.

### Estimates of Fixed Effects and Odd Ratios for CBM

The effects estimated in CBM are on a liability scale. The estimated effect of sex (being female) was -0.26± 0.04 (p-value<1e-6), with an odds ratio of 0.77, implying a decreased risk of developing diabetes in females, relative to males. The estimated effect of the Offspring cohort was -0.40±0.06 (p-value<1e-6), with an odds ratio of 0.68, implying a lower risk of diabetes for subjects on the Offspring cohort relative to subjects of the Original cohort. This estimate is probably affected by the shorter observational period of the members of the Offspring cohort, even though the age at the last contact was included in the study to correct for different censoring times. The estimated effect for the age at the last contact was 0.016±0.002 (p-value <1e-6) with an odds ratio of 1.02, implying that there is a slightly higher risk of observing diabetes per year of exposure. Finally, PCs 1 and 2 were non-significant with odds ratios of 1.25±1.89 (p-value = 0.57) and 1.18±2.06 (p-value = 0.57), respectively. However, in order to correct for population stratification, we retained the principal components in the model.

### SNP Effects, Genetic Effects and Genetic Parameters

Odds ratio for the 65 SNPs obtained with the M-65SNP model and odds previously reported in the literature [30] have a correlation of 0.42. Genetic scores were calculated for all subjects with the different models. The genetic risk score derived with the G-BLUP model ranged from -0.980 to 1.766 and was centered at -0.003±0.498 (mean ± standard deviation). The genetic risk score with G-BLUP, and M65-SNPs had a positive but weak association of 0.23. The much higher amount of SNPs between the M-65SNPs and the G-BLUP brings different information to the prediction of genetic scores.

**Fig 2:**
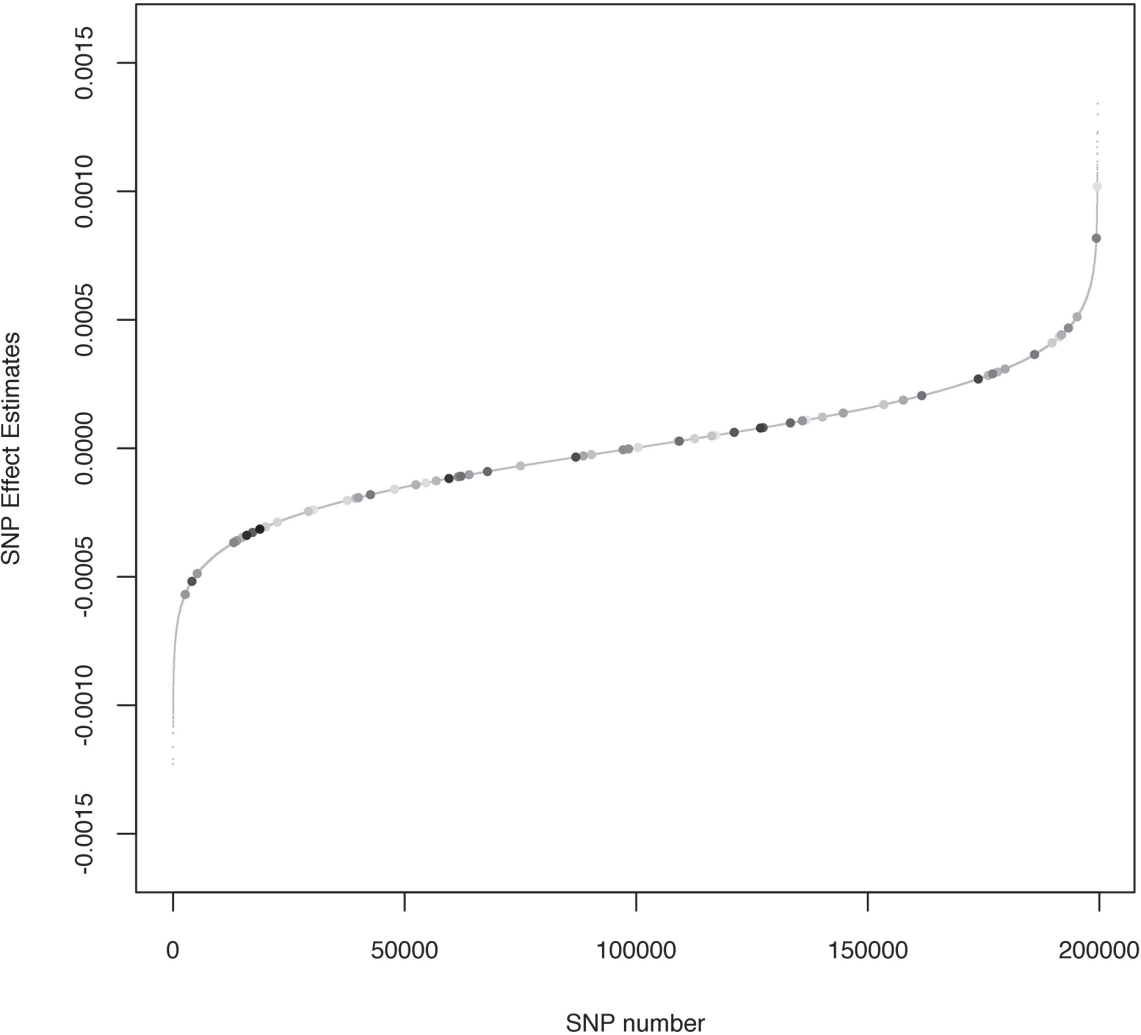
SNP estimated effects ordered by effect for G-BLUP. This is re-parameterized with a Bayesian Ridge Regression. Dots show the effects of the 65 SNPs are and are on a gray scale; the darker the dot, the more significant is its association with the response.

Fig 2 shows the estimated SNP effect with G-BLUP model (re parameterized as a Bayesian Ridge Regression) and the dots highlight where the effects of the 65 SNPs of the M-65SNPs model are. It can be observed that the highly significant markers were not always the SNPs with the greatest effect. Models BC and BLASSO produced estimated effects of the SNP with similar shrinkage than one observed in Fig 2; BA however had convergence problems in the full data analysis.

**Fig 3:**
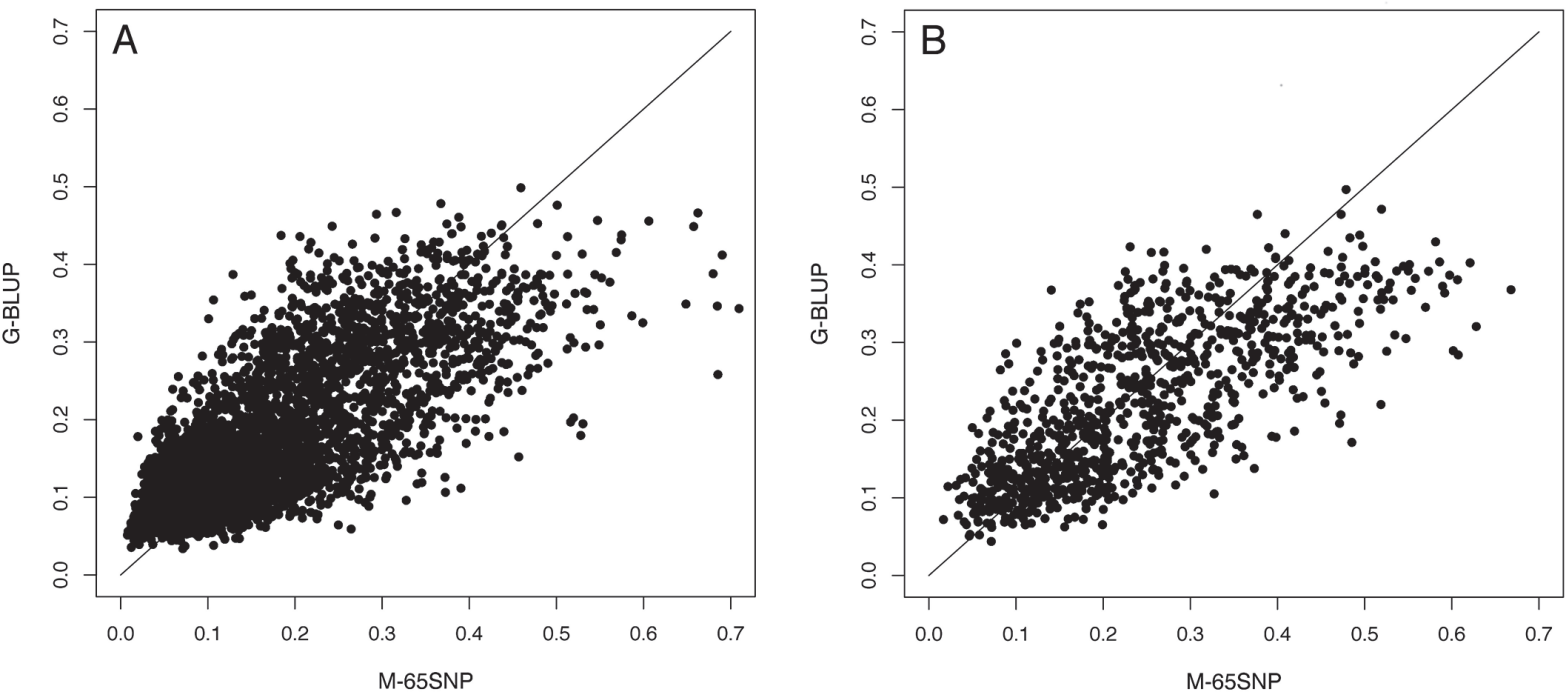
Probability of diabetes for M-65SNP and G-BLUP. These are classified by the presence or absence of diabetes: a) healthy and b) diabetic people.

Fig 3 shows the relationship between the probability of having diabetes for healthy (a) and diabetic (b) participants. Estimates in the scatter plots were obtained from the G-BLUP model and M-65SNPs. Other WGR (not shown) presented a strong association with the probabilities estimated with the G-BLUP. The line is a slope of one, indicating the hypothetical situation in which both models estimate the same probability of having diabetes for a person. M-65SNPs is based on fixed-effect estimates. For this model, we observe that each SNP has stronger effect than the same SNP in the G-BLUP model because a fixed effect is a value close to the conditional mean of all the samples with that SNP (jointly with deviations given by other factors); while the RKHS predicts random effects where the SNP effect is pushed towards zero. The resulting estimates for a person that has all the high-risk SNPs (of the 65) accumulate into a very large estimated overall risk, while in the RKHS, the estimated effects of these SNPs are mitigated and predicted risk is thus lower.

#### Bayes C*π* estimates

The estimated proportion of SNPs with a genetic signal (1- π) was quite large at 0.579± 0.114. Thus, this result suggests that more than 50% of the total number of SNPs contributed to the overall genetic variation, although the individual contribution of most of these SNPs is likely to be a miniscule proportion of the total genetic variation.

#### Genomic Heritability of Type 2 Diabetes

The G-BLUP model results in an estimate of genetic variance associated with common SNPs in the full training sample of 0.92 ± 0.26. Considering that the residual variance was fixed at 1, the estimate of genomic heritability for Type 2 Diabetes was 0.492±0.066. Previous studies demonstrated that in family datasets genomic heritability from G-BLUP yielded similar results [3,21].

### Predictive Ability of the Models

Models were also evaluated for their predictive ability using a 10-fold cross-validation. We randomized families and assigned entire families to folds. The number of families per fold and the size of the families can be seen in S1 Table. The number of subjects per fold was between 364 and 727 individuals, contained within 147 to 181 families.

Table 1 shows the predictive AUC of the models (in the testing sets of the cross validation). The predictive ability of all the models that included genetic information improved with respect to the model only with covariates. However, the improvement was moderate and there were not important differences between models.

**Table 1:** Area Under the Receiver Operating Characteristic Curve (AUC) for the CBM, M-65SNP, BRR, BA, BC, B-LASSO and G-BLUP.

Model	AUC-CV (Mean ± S.D.)
CBM	0.668 ± 0.025
M-65SNP	0.684 ± 0.041
BA	0.678 ± 0.027
BC	0.680 ± 0.027
B-LASSO	0.681 ± 0.027
G-BLUP	0.678 ± 0.027

## Discussion

Diabetes increases morbidity and mortality significantly, even when the condition is treated [51]. Diabetes is expected to rise from 171 million in 2001 (globally) to 366 million by 2030 [52]. Thus, it is imperative that we understand the underlying causes of diabetes, enhance our ability to identify those at risk, and mitigate that risk. We found that both genetic and non-genetic factors are associated with diabetes. In the Framingham study, gender is associated with developing T2D and has an odds ratio of 0.77 for females. Age also clearly matters, as evidenced in our study by the odds ratio of 0.68 for the Offspring cohort (that is younger) and the odds ratio of 1.02 per year of exposure for the effect of age at last contact. The Framingham cohorts are primarily Caucasian and are of European descent [53] (see Fig 1), and T2D susceptibility did not vary with ancestry in this population. This is contrary to findings for conditions such as skin cancer, which may be influenced by differences in ancestry even among those of European descent [54] and may indicate that risk alleles for T2D among those of European descent are relatively homogeneous. Although diabetes prevalence is not consistent across ethnicities, we found no evidence of origin differences within Caucasians. Genetic factors also appear to play a substantial role in T2D susceptibility. In our paper we estimated a genomic heritability which is within the range of what has been reported in the literature. Additionally, there are several reports of uncovered SNP variants associated with diabetes, and in our study we confirmed evidence of association between individual genetic score and T2D.

The predictive ability of the WGR models was moderately improved in comparison to the model based only on clinical covariates. It is known that prediction accuracy will greatly depend on the genetic distance between training and testing sets [55–57]. Several animal and plant studies [17,43,55,58]; [59,60] and some human studies where training and validation samples are closely related [18,20,61] have shown that WGRs can achieve high predictive power and sometimes even produce better predictions than those based on pedigrees [54]. For unrelated individuals, theoretical formulae for the prediction accuracy of G-BLUP [62,63] suggest that achieving reasonably good accuracy of estimates of effects for dense marker panels will require using extremely large samples. According to [62] the prediction accuracy of a WGRs depends on two main factors: (a) the proportion of variance that can be explained by regression on markers, and (b) the accuracy of effects estimates. As more markers are added to a model, genomic heritability increases; however, the more markers we include in the model the lower the accuracy of estimates of individual effects. Consequently, for any given sample size, adding large numbers of markers to the regression may increase the estimated genomic heritability but will not necessarily result in higher predictive power.

In our study, most models evaluated did not differ in the shrinkage of the SNP estimates or in the prediction accuracy. In agreement with our results in humans, Daetwyler and coauthors in a review paper report that there are not major differences in the predictive ability of WGR methods in several animal and plant studies [22]. Most traits of interest are highly complex and the benefit of heavy tailed distribution or mixtures is expressed more in traits where few genes explain a sizable proportion of the genetic variance [40]. Our results suggest a highly complex genetic architecture of T2D; in this situation there are no markers that could improve the modeling by capturing a greater signal since there is no greater signal to be captured. Models that are able to do selection selected approximately 50% of the markers. However, our result may also be affected by population substructure in the training sample since the training set has families. In these samples of related subjects, causal SNPs will be in long regions of high LD, whereas in samples with unrelated subjects these regions will be shorter; thus, within that region any SNP would be an equally good predictor to capture the effect of the causal SNP. Consequently, this ‘more complex’ architecture mitigates differences between the models. Having training sets with families is equivalent to reducing the sample size of the training set, which we know is essential to achieve good prediction accuracy[43,62]. Still, among all the methods, the Bayesian LASSO had slightly thicker tails, allowing for more markers to have higher effects, while also achieving higher prediction accuracy.

Nevertheless, the AUC and performance achieved with 65 known markers was the same as the one achieved with Bayes C*π* and the G-BLUP. In the case of diabetes, BMI, height, and several other complex traits or diseases, mega consortiums are pulling thousands of genotyped subjects and their phenotypes to find the SNPs highly associated with the phenotype. However, for several phenotypes these resources are not available, and will probably never be (e.g., rare diseases), and significant SNP markers are unknown. Hence it is worth using the G-BLUP or Bayes C*π*, both of which achieved the same performance relative to the model using information of the well-established SNPs without using any prior information from large GWAS consortiums.

In summary, we found evidence of genetic effects in T2D, which reflects in the heritability estimates; additionally, existing genetic variation can be captured by high-density markers. Results from different WGR methods did not differ. We can find similar reports in the animal science literature; the improvement in AUC using cross validation was positive, albeit poor. Finally, the AUC from 65 well-known variants affecting diabetes was similar to that obtained from including all variants. However, these 65 SNPs were found in a large consortium study. Most complex traits and diseases do not have large consortiums data available, and in rare diseases, it is possible that there will never be large amounts of phenotypic and genomic data. WGRs could be important for diseases where no large consortium is available. Bayes LASSO and G-BLUP models are alternative methodologies using dense arrays of markers.

## Supporting Information

S1 Fig Trace plot and density plot of the genetic variance associated to the markers in the dense SNP array. (TIFF)

S2 Fig Trace plot and density plot of the genetic variance associated to the markers in the dense SNP array. (TIFF)

S1 Table Number of families and subjects assigned to each fold of the 10-fold cross-validation. (DOCX)
