# Supplementary figures and images for: Assessment of Whole-Genome Regression for Type II Diabetes
Source: PLoS One. 2015 Apr 17;10(4):e0123818. doi: 10.1371/journal.pone.0123818 (PMC4401705; doi:10.1371/journal.pone.0123818)

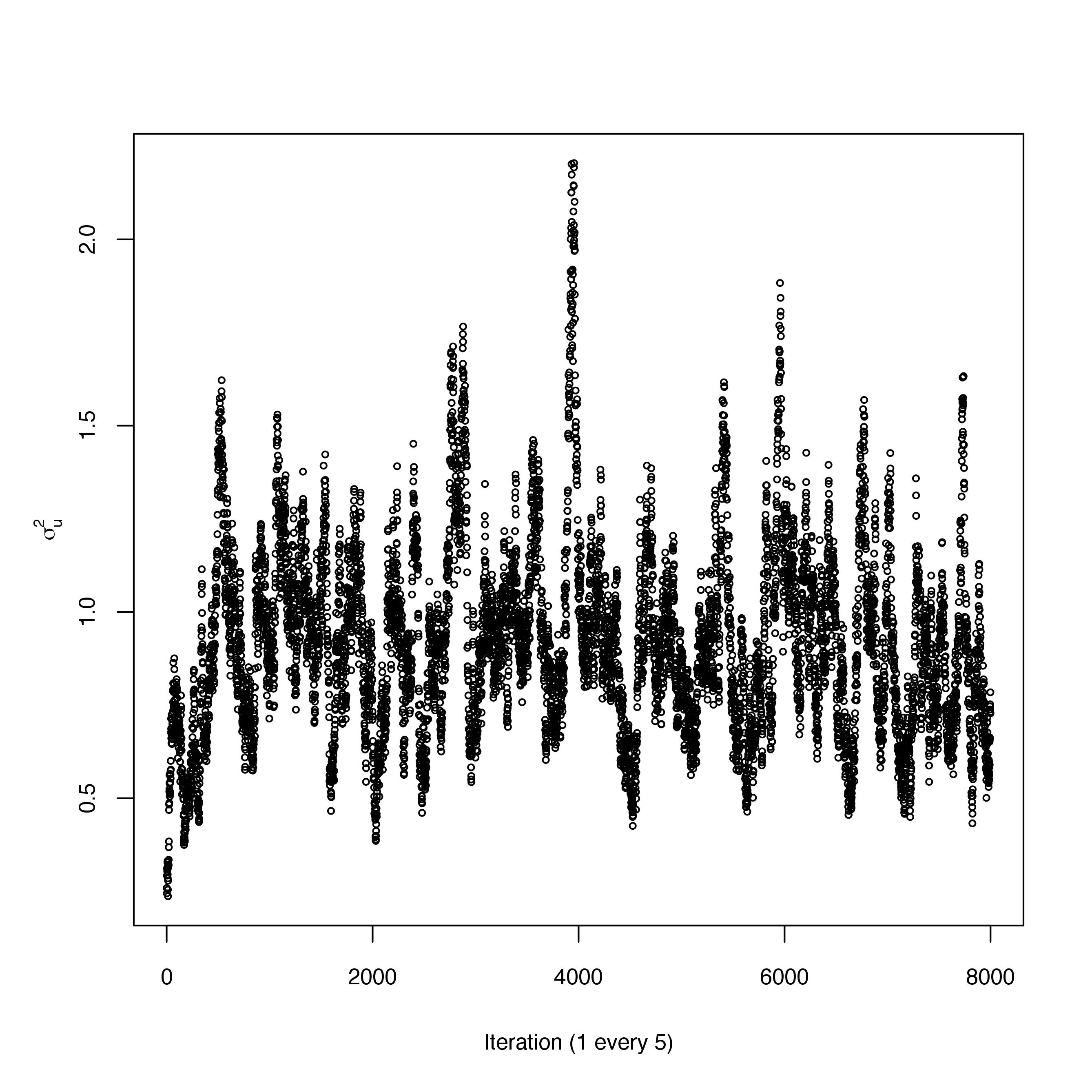

Supplement: S1 Fig — (TIFF) [file pone.0123818.s001.tiff]

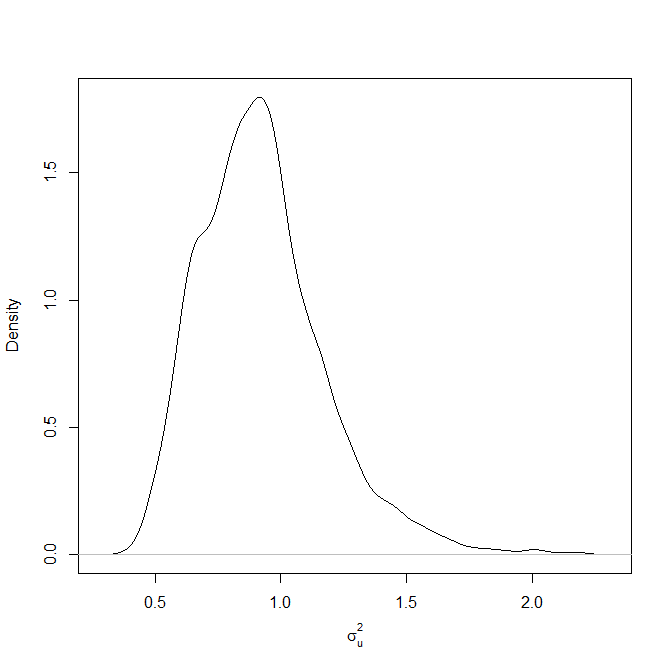

Supplement: S2 Fig — (TIFF) [file pone.0123818.s002.tiff]
