# Supplementary material for: Assessment of Whole-Genome Regression for Type II Diabetes
Source: PLoS One. 2015 Apr 17;10(4):e0123818. doi: 10.1371/journal.pone.0123818 (PMC4401705; doi:10.1371/journal.pone.0123818)
Supplement: S1 Table — (DOCX) [file pone.0123818.s003.docx]

| **Fold** | **# Subjects** | **# families** |
| --- | --- | --- |
| 1 | 678 | 186 |
| 2 | 498 | 163 |
| 3 | 517 | 183 |
| 4 | 462 | 153 |
| 5 | 382 | 157 |
| 6 | 504 | 151 |
| 7 | 645 | 163 |
| 8 | 364 | 147 |
| 9 | 468 | 172 |
| 10 | 727 | 181 |
